# Selection Criteria and Treatment Outcome for Advanced Non-Small Cell Lung Cancer (NSCLC) Patients Unfit for Platinum-Based First-Line Therapy: Results of the MOON-OSS Observational Trial

**DOI:** 10.3390/cancers14246074

**Published:** 2022-12-09

**Authors:** Andrea Camerini, Alessandro Del Conte, Aldo Pezzuto, Vieri Scotti, Francesco Facchinetti, Lucia Pia Ciccone, Marco Perna, Giulia Sartori, Cheti Puccetti, Alberto Ricci, Antonio Santo, Marcello Tiseo, Domenico Amoroso

**Affiliations:** 1 Medical Oncology, Versilia Hospital, Via Aurelia 335, 55043 Lido di Camaiore, LU, Italy; 2 S.O.C. Oncologia Medica e dei Tumori Immunocorrelati—CRO/IRCCS Aviano Via Gallini 2, 33081 Pordenone, PN, Italy; 3 Cardiovascular and Respiratory Sciences Department, Sant’Andrea Hospital, Via di Grottarossa 1035/1039, 00189 Roma, Italy; 4 Radiation Oncology Unit, Oncology Department, AOU Careggi, Largo Brambilla 3, 50134 Firenze, Italy; 5 Institut Gustave Roussy, Inserm, Biomarqueurs Prédictifs et Nouvelles Stratégies Thérapeutiques en Oncologie, Université Paris-Saclay, 114 Rue Vaillant, 94800 Villejuif, France; 6 Medical Oncology Unit, University Hospital of Parma, Viale Gramsci 14, 43126 Parma, Italy; 7 Section of Medical Oncology, Department of Medicine, University of Verona, Piazzale Stefani 1, 37126 Verona, Italy; 8 Department of Clinical and Molecular Medicine, Università La Sapienza, Via di Grottarossa 1015, 00189 Roma, Italy; 9 Lung Unit, Ospedale Pederzoli—Via Monte Baldo 24, 37019 Peschiera del Garda, VR, Italy; 10 Department of Medicine and Surgery, University of Parma, Viale Gramsci 14, 43126 Parma, Italy

**Keywords:** elderly, low performance status, single agent chemotherapy, non-small cell lung cancer, metronomic

## Abstract

**Simple Summary:**

The treatment of advanced NSCLC patients unfit for a platinum combination is challenging and no clear evidence is available. We retrospectively collected data on advanced NSCLC not receiving a first-line platinum combination, focusing on clinical selection criteria. Up to 30% of newly diagnosed advanced EGFR/ALK negative and PD-L1 < 50% NSCLC patients do not receive a first-line platinum doublet. Main clinical selection criteria were older age (>70 years), comorbidities and poor performance status. A single agent chemotherapy was offered with the prevalence of oral metronomic vinorelbine. In the whole population, progression-free survival and overall survival ranged from 4.5 to 5 months and from 9 to 10.5 months, respectively.

**Abstract:**

Limited evidence is available concerning the selection criteria and the outcomes of platinum unfit newly diagnosed advanced NSCLC patients receiving single-agent chemotherapy. We retrospectively collected data on consecutive, stage IIIB-IV, EGFR/ALK negative and PD-L1 < 50% NSCLC patients treated with first-line single agent chemotherapy. Baseline characteristics, outcome measures and toxicities were recorded, as well as criteria according to which treatment selection was made and what percentage of patients did not receive a first-line platinum-based chemotherapy. Two-hundred and twenty-one patients were included. Median age was 79 (range 56–92) years, M/F 165(74.6%)/56(25.4%), ECOG performance status (PS) 0/1/ ≥ 2 23(10.9%)/94(42.5%)/103(46.6%), with a median of two serious comorbidities. A median of 25% (range 10%-30%) of newly diagnosed NSCLC did not receive a first-line platinum combination. Clinical criteria according to which decision was made were older age (76.5%), comorbidities (72%), poor PS (55.2%) and familiar or social issues (10%). Single-agent treatment consisted of oral metronomic vinorelbine (MetV 78.6%), gemcitabine (Gem 10%), oral standard vinorelbine (Vin 8.2%) and other (O 3.2%). Median progression-free survival (PFS) and overall survival (OS) of single agent treatments ranged from 4.5 to 5 months and from 9 to 10.5 months, respectively. All grade toxicities did not differ among single agents, while grade 3–4 toxicities were less frequent with MetV. Up to 30% of newly diagnosed advanced EGFR/ALK negative and PD-L1 < 50% NSCLC patients do not receive a first-line platinum doublet. Main clinical selection criteria were older age (>70 years), comorbidities and poor PS. An oral treatment was frequently proposed with MetV being the most frequent choice according to its safety profile.

## 1. Introduction

Non-small cell lung cancer (NSCLC) is the leading cause of cancer death in Western World [1,2]. More than 50% of cases of advanced NSCLC are diagnosed in patients older than age 65 years, and approximately 30% to 40% in patients older than age 70 years [3,4]. The standard first-line treatment for advanced NSCLC without actionable oncogenic drivers and with programmed death-ligand 1 (PD-L1) tumor proportion score (TPS) < 50% is a palliative doublet platinum-based chemotherapy combined with immunotherapy (if not contraindicated) [5]. Not all patients are fit for such regimens. The assessment of frailty is still underperformed and needs dedicated and validated tools even in the immunotherapy era [5,6,7]. The definition of unfit patients harbors a significant degree of subjectivity and varies among clinicians. In 2015, criteria were proposed to define fitness, including age, ECOG PS, renal function, heart failure, previous cerebrovascular events, uncontrolled hypertension, neuropathy, hearing loss, symptomatic brain metastases, severe psychiatric disorders and the absence of caregiver support [8]. Nevertheless, its backbone is constituted by two conditions: age and ECOG performance status (PS). The patients could be unfit for cisplatin and/or carboplatin combined chemotherapy [8]. For patients unfit for both platinum agents (thereafter referred to as “platinum-unfit”), single-agent chemotherapy is a valid treatment option [5]. Several drugs showed activity as monotherapy in NSCLC like vinorelbine, gemcitabine, pemetrexed and taxanes. The relevance of age and ECOG PS in first-line treatment allocation has been explored by Corre et al., highlighting the relevance of these parameters in outcome and toxicity prediction [9]. The high incidence of advanced NSCLC in the elderly, coupled with the relevant number of comorbidities (frequently related to smoking habit) and the high symptom burden of lung cancer make the diagnosis of NSCLC in unfit patients a frequent occurrence. Given the lack of a universal definition of “unfit” and the underrepresentation of elderly and low PS patients in clinical trials even to date, an estimate of the real percentage of unfit patients approaching first-line treatment is difficult. Indirect data indicate that a range from 15% up to 30% of first-line NSCLC patients can be deemed unfit for platinum combos [10].

The aims of the present study were to assess what percentage of first-line patients do not receive a platinum combination, to investigate on treatment selection criteria when a single-agent first-line therapy was delivered, and to collect data on patient outcomes and treatment safety.

## 2. Patients and Methods

### 2.1. Study Population

MOON-OSS was an Italian multi-center retrospective observational trial. We collected data of hystologically or cytologically confirmed, Epidermal Growth Factor Receptor (EGFR) and Anaplastic Lymphoma Kinase (ALK) negative, PD-L1 < 50%, stage IIIB (not suitable for surgery and chemo-radiotherapy) or IV NSCLC according to UICC-TNM 7th edition. No restrictions based on histology were applied. To be enrolled, all participants should have been deemed not fit to receive a platinum combination by a treating physician and so being treated with any first-line single agent from 2015 to 2017 outside a clinical trial. The inclusion criteria of tumor proportion score (TPS) PD-L1 expression lower than 50%, even if not relevant in treatment selections in Italy at that time, has been introduced to make our study population comparable to actual clinical practice taking into consideration the results of the KEYNOTE 024 trial [11] available at that time. PD-L1, EGFR and ALK evaluation have been performed in local referral laboratory according to standard procedure and no centralized testing was performed. Patients concomitantly receiving any other anticancer agent were excluded. Adjuvant treatment represented an additional exclusion criteria. Patients received any other palliative treatment needed as per local practice. Investigators were asked to compare the number of study patients to the global amount of first-line advanced EGFR/ALK negative and PD-L1 < 50% NSCLC that received an active treatment in the same time period.

Patients’ names were coded and not revealed. All relevant clinical, radiological and pathological information were recorded in an appropriate database. Clinical records were extracted and collected locally by investigators and survival data were collected from registry office if necessary. The study received approval by local ethical committee (Approved on 3 October 2016 by Comitato Etico Area Vasta Nord Ovest, Regional ref. number 527, Prot. 0167006). Informed consent was demanded for patients alive at the time of data recording.

### 2.2. Endpoints and Assessment

The primary objectives of the study were to define the percentage of first-line advanced NSCLC patients without actionable driver mutations (EGFR/ALK), and with an expression of TPS PD-L1 < 50%, that did not receive a platinum combination, to describe the selection criteria according to which that choice has been made, and to collect data on the type of single agent selected.

Secondary objectives included time progression-free survival (PFS), PFS without grade 3/4 toxicity (G3/4PFS), overall survival (OS), overall response rate (ORR), disease control rate (DCR) and toxicity.

Disease response to treatment was assessed according to RECIST 1.1 [12]. Disease control rate (DCR) was the sum of complete and partial responses and disease stabilization lasting more than 12 weeks. Progression free survival was calculated from the date of treatment initiation to the date of the first-documented progression and G3/4PFS was calculated from treatment start until the date of progression, or the date of grade 3/4 toxicity (or the date of death due to any cause, whichever occurred first for both). Survival was measured from the date of treatment initiation up to death or last follow-up. For patients who had not died survival time was censored at the date of last news.

Disease evaluation during treatment was performed as per local practice. Adverse events were recorded according to the Common Terminology Criteria for Adverse Events (CTCAE) v4.3 [13]. We consider the following as serious comorbid illnesses: heart disease (previous myocardial infarction, heart failure, valvular heart disease and serious arrhythmias), chronic obstructive pulmonary disease (COPD), diabetes, cerebral or peripheral vascular disease, chronic renal failure, hepatitis and/or cirrhosis, hypertension and severe auto-immune disorders.

### 2.3. Statistical Analysis

Given the descriptive aim of the study, no formal statistical design was set up. Descriptive data are presented as percentages of the whole patient number, while survival data and number of delivered cycles are presented as median parameter with range. Given the retrospective design of the study and the non-homogenous distribution of patients in treatment groups no statistical test was performed to compare survival between different groups.

## 3. Results

### 3.1. Baseline Characteristics

Overall, 221 EGFR/ALK negative and PD-L1 < 50% advanced NSCLC patients unfit for a platinum combination were enrolled from January 2015 to June 2017. Baseline patients’ characteristics are reported in Table 1. The majority of patients were male (n = 165/221 74.5%), current or former smokers (n = 60/221 27.1% and n = 138/221 62.5%, respectively) with stage IV disease (n = 159/221 71.9%). Main histology was adenocarcinoma (n = 107/221, 48.4%) but squamous cell carcinoma was relevantly represented as well (n = 94/221 42.5%). The unusual high percentage of squamous histology (usually less than 30%) could be related to the high proportion of male and current or former smokers in the study population. The study population can be undoubtedly defined as elderly with a median age of 79 (range 56–92) years. Almost half of the patients presented with an ECOG PS of two or more (n = 103/221 46.6%) and a median of two serious comorbidities (mainly hypertension and COPD). Disease burden was relatively high in our study population with a median of three disease sites. More frequent disease sites were lung (both as primary site in stage IIIB and as metastatic site in stage IV), pleura, mediastinal lymph-nodes and bone.

### 3.2. Selection Criteria, Treatment Disposition and Outcome

**Figure 1:**
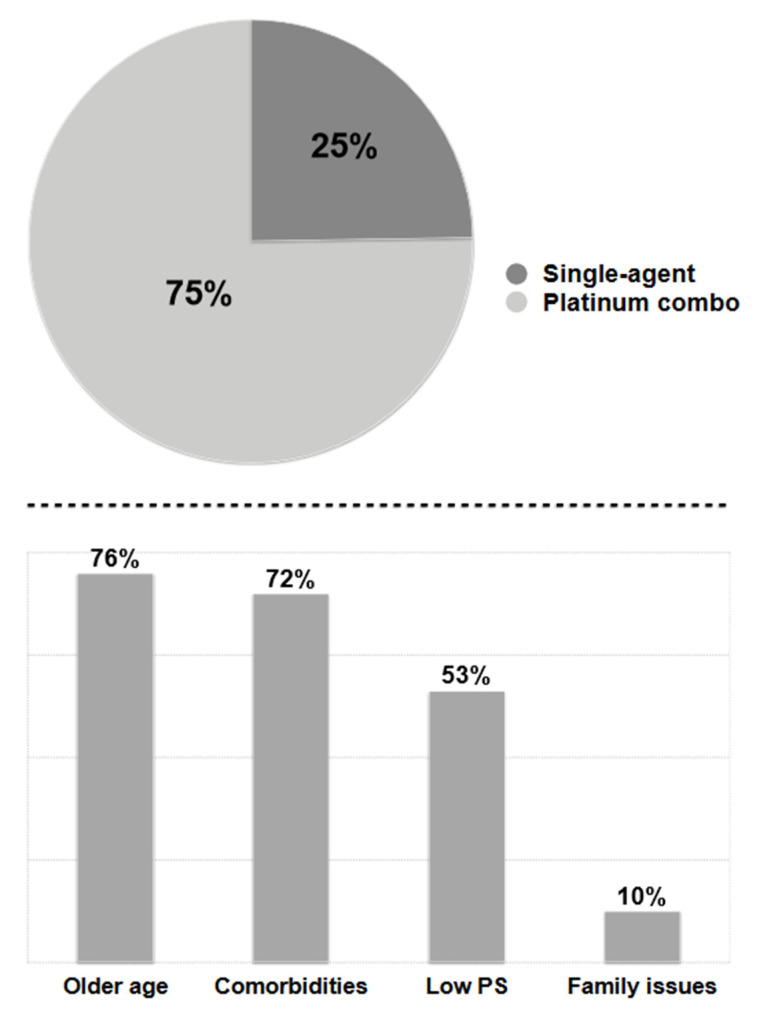
Treatment disposition related to platinum use in first-line EGFR/ALK negative and PD-L1 < 50% advanced NSCLC (upper panel) and main reasons to avoid platinum (lower panel).

Taking into consideration the global number of patients with study characteristics (TPS PD-L1 < 50% and without EGFR and ALK driver target mutations) receiving an active first-line treatment during study period (approximately 30 months), 25% (range 10–30%) were deemed platinum unfit and accordingly did not receive a platinum combination (Figure 1, upper panel). Physicians were asked about the reasons for their choice and the main clinical selection criteria were older age (n = 169/221 76.5%), comorbidities (n = 159/221 72%), poor (≥2) ECOG PS (n = 122/221 55.2%) and familiar or social issues (n = 22/221 10%) (Figure 1, lower panel). Multiple reasons were referred in the majority of cases (n = 195/221 88.2%) and rarely a single item was responsible for the choice to avoid platinum (n = 26/221 8.6%). In this latter group (n = 26), extremely older age (≥85 years) (n = 13/26 50%) and poor ECOG PS (n = 13/26 50%) were the single items guiding the choice.

Single-agent treatment consisted of oral metronomic vinorelbine (n = 174/221 78.6%), gemcitabine (n = 22/221 10%), oral standard vinorelbine (n = 18/221 8.2%), and other (pemetrexed, docetaxel) (n = 7/221 3.2%) (Table 1).

Median PFS did not show significant differences among the four groups of single agent treatment, ranging from 4.5 to 5 months. A similar situation was observed in overall survival (OS) with no single agent showing a clear survival advantage. Overall survival ranged from 9 to 10.5 months. Single agent treatments showed an interesting DCR ranging from 60% to 77% with an ORR around 15% without significant differences among treatments.

Given the challenging patient population with a higher risk for toxicity and for premature treatment discontinuation due to treatment-related side effects, a special focus on toxicity-free survival was set by collecting data on both PFS and OS without grade 3 or 4 toxicity. Median PFS and OS without grade 3 or 4 toxicity ranged from 4.5 to 6.5 months and 10 to 12 months, respectively.

### 3.3. Safety

Probably, treatment related side effects are the major concern of physicians when planning an active treatment in platinum-unfit patients. Globally, serious treatment-related adverse events were less frequently experienced by patients treated with metronomic oral vinorelbine (8%) compared to gemcitabine (13.6%), oral standard vinorelbine (16.6%) and others group (14.3%). All-grade toxicities did not show relevant differences in incidence among single agent treatments. According to the lower level of grade 3 or 4 toxicity with oral metronomic vinorelbine, both dose delays (gemcitabine 41%, oral standard vinorelbine 16.6%, metronomic vinorelbine 13.8%, others 28.6%) and dose reductions (gemcitabine 31.8%, oral standard vinorelbine 33.3%, metronomic vinorelbine 17.8%, others 28.6%) were less frequent with metronomic administration (Supplementary Table S1).

## 4. Discussion

Treatment selection in elderly and/or poor performance status patients still represents a tough choice. Often, advanced NSCLC patients present with highly symptomatic disease at diagnosis time and a rapid worsening of general health status is not uncommon. A relevant percentage of elderly newly diagnosed metastatic NSCLC patients is not suitable for any active treatment and received best supportive care (BSC). Seto et al. reported a percentage of 60% (75 patients out of 124) of elderly (aged ≥75 years) people with mainly advanced NSCLC being treated with BSC alone. The main reasons for receiving BSC were poor PS, advanced age together with dementia and the patients’ wish. Interestingly, single agent chemotherapy was the selected treatment in the majority of cases receiving an active treatment [14]. Similar data were reported in a retrospective analysis about the reasons for choosing BSC as initial treatment. Serious comorbidities, poor ECOG PS and social background were the determinants of BSC, but the percentage of patients not receiving an active treatment reduced to 12% [15]. Data from a large retrospective observational study in the US reported that 76% of advanced NSCLC did not receive any active treatment at diagnosis. The percentage of patients who received chemotherapy increased slightly but significantly over time, from 19% in 2005 to 26% in 2009, and patients older than 70 years were significantly less likely to receive treatment with chemotherapy (*p* < 0.0001). The preferred first-line chemotherapy was a platinum doublet but 11% received single agent treatment [16]. Confirmatory data came from a meta-analysis on 38 trials of advanced NSCLC from 1991 to 2011. The authors reported that male gender, poor performance status, as well as distant metastases and recent weight loss predict for poor OS in older patients [17].

Our data showed that a relevant proportion, up to 30%, of patients with advanced NSCLC did not receive a platinum combination. Study population characteristics should be taken into consideration when analyzing the percentage of patients not receiving platinum. In fact, the median age of near 80 years old, together with the unusually high percentage of squamous histology and the prevalence of current or former smokers, limited the use of more toxic combinations as those platinum-based. The median number of serious comorbidities overlaps with other reports in locally advanced or metastatic settings [18,19]; in our population, smoking-related conditions such as hypertension, COPD, heart disease and cerebral or peripheral vascular disease presented with a high incidence, and these comorbid illnesses are of particular concern for clinicians, and so their presence influenced the decision of not to deliver platinum. The study participating centers were both non-teaching and university hospitals, set on urban and rural areas and spread throughout Italy, so data cannot be influenced by hospital type, and describe the real prescribing attitude of physicians. Of note, centers were selected on the basis of their expertise to treat lung cancer, so an even higher percentage of unfit patients in non-specialized centers is likely. A real-world analysis of hospital-based cancer data on advanced NSCLC with no driver mutations in Japan pointed out that platinum doublets were prescribed for 62.7% of the patients aged >70 years and for 60.7% of those >75 years in NSCLC, and 37.6% received single agents [20].

Unfit definition is the threshold between platinum combinations or single agent treatment, and the decision is up to the physician. Reported data clearly indicate that treatment selection and “platinum fitness” is a multi-parametric choice. Unfit definition is based on a single parameter in only 8% of cases and so in the large majority of cases it took into consideration different clinical items. Older age, low ECOG PS and comorbidities confirmed to be the priorities in treatment selection in accordance with the clinical definition of platinum fitness in the aforementioned proposal [8] and propose a streamlined approach suitable in clinical practice. The clinical items on which treatment selection is based in our experience must not be considered as an alternative to validated tools as comprehensive geriatric assessment (CGA); they represent an additional help when CGA is not feasible or in uncertain situations.

Study data on single agent efficacy in this challenging clinical scenario are in accordance with previous experiences. The majority of patients received metronomic therapy with oral vinorelbine (78.6%) confirming this approach as one of the most common in clinical practice. All observed efficacy and safety parameters of metronomic vinorelbine overlap with previous published data. Prospective phase II data and large international real world evidences highlighted the safety profile of metronomic vinorelbine coupled with an interesting clinical activity [21,22]. The random Tempo-Lung trial has proven in unfit patients with advanced NSCLC the superiority of first-line metronomic vinorelbine over standard oral vinorelbine in terms of PFS without grade 3 or 4 toxicity and confirmed the excellent safety profile [23]. For the sick of clarity, no data is available (in our study or on medical literature) on a comparison between metronomic vinorelbine and platinum combination, so no conclusion can be made. 

Recently, data of the IPSOS trial have been presented but not yet fully published [24]. This trial showed a modest but statistically significant survival advantage for first-line atezolizumab over single agent chemotherapy in platinum unfit patients. The population was not selected on the basis of PD-L1 expression level, so it can be argued that a proportion of high PD-L1 expressors could be present in atezolizumab arm, so explaining (at least in part) the observed difference. Moreover, no subgroup analysis is available on the relative efficacy in the two main subgroups (elderly and PS 1 or PS 2/3) and this could also influence efficacy. Interestingly, an all-immunotherapy combination of nivolumab plus ipilimumab did not show any survival advantage in PS2 patients over a platinum combination [25].

As a direct consequence of unfit definition, patients excluded from platinum combinations are also ruled out from combined chemo-immunotherapy treatments. Poor PS and elderly patients, as well as patients with serious comorbidities, have not been enrolled in pivotal chemo-immunotherapy trials so no data on these challenging populations are available [26,27]. On the other hand, older age per se seemed not be an absolute contraindication to immunotherapy combinations with platinum [28], but a definitive conclusion cannot be drawn. Our findings pointed out that the definition of platinum-fitness is mainly based on PS, age and comorbidities, hence “unfit” patients should be directly excluded from such combination treatments. An interesting development is represented by the possibility to combine immunotherapy to single agent chemotherapy. The preliminary results of the run-in part of a single arm phase II the combining atezolizumab plus oral metronomic vinorelbine in pre-treated advanced NSCLC showed the feasibility of the combination [29]. Alternatively, a mathematical modeling of metronomic vinorelbine has been proposed [30,31].

## 5. Conclusions

The MOON-OSS trial provided an overview on treatment patterns of advanced NSCLC deemed not fit to receive a platinum-based combination. We confirmed that a relevant percentage (up to 30%) of newly diagnosed advanced EGFR/ALK negative and PD-L1 < 50% NSCLC patients do not receive a first-line platinum doublet. The decision not to deliver platinum is based on a combination of clinical criteria as older age (>70 years), comorbidities and poor (≥2) PS, while social and familiar issues are less relevant. In such a challenging population, single agent oral treatment is frequently proposed. The MOON-OSS retrospective real world observations need to be confirmed on a larger sample size and provided the rationale for a prospective observational trial aimed to confirm the role of clinical selection in treatment disposition.

## Figures and Tables

**Table 1:** Patients’ demographic, clinical-pathological characteristics and treatment disposition.

Characteristics (Total n = 221)	n (%)
**Age median (range)**	79 (56–92) years
**Sex**	
Female	56 (25.5%)
Male	165 (74.5%)
**Smoker status**	
Current smokers	60 (27.1%)
Former smokers	128 (62.5%)
Never smokers	33 (10.4%)
**Disease stage**	
IIIB	62 (28.1%)
IV	159 (71.9%)
**ECOG PS**	
0	23 (10.9%)
1	94 (42.5%)
≥2	103 (46.6%)
**Histology**	
Adenocarcinoma	107 (48.4%)
Squamous cell carcinoma	94 (42.5%)
Large-cell carcinoma	9 (4.1%)
NOS carcinoma	11 (5%)
**Comorbid illnesses median (range)**	2 (0–6)
Comorbid illnesses *	
Hypertension	166 (75.1%)
COPD	141 (63.8%)
Heart disease	120 (54.3%)
Cerebral or peripheral vascular disease	95 (43%)
Diabetes	66 (29.9%)
Chronic renal failure	52 (23.5%)
Hepatitis and/or cirrhosis	24 (10.9%)
Severe auto-immune disorders	15 (6.8%)
**Disease sites median (range)**	3 (1–5)
Disease sites *	
Lung °	138 (62.4%)
Lymph nodes	112 (50.7%)
Pleura	108 (48.9%)
Bone	95 (43%)
Adrenal	88 (39.8%)
Liver	35 (15.8%)
Brain	22 (9.9%)
Other	15 (6.8%)
**Single-agent treatment**	
Gemcitabine	22 (10%)
Oral standard vinorelbine	18 (8.2%)
Metronomic vinorelbine	174 (78.6%)
Others *	7 (3.2%)

ECOG PS, Eastern Cooperative Oncology Group performance status. NOS, non-otherwise specified. COPD, chronic obstructive pulmonary disease. * Multiple comorbidities/disease sites in a single patient. ° As primary site in stage IIIB and as metastatic site in stage IV.

## Data Availability

The data presented in this study are available on request from the corresponding author. The data are not publicly available due to institutional policy.

## Supplementary Materials

The following supporting information can be downloaded at: https://www.mdpi.com/article/10.3390/cancers14246074/s1. Table S1: Safety summary of single agent treatments.
