# Supplementary material for: Selection Criteria and Treatment Outcome for Advanced Non-Small Cell Lung Cancer (NSCLC) Patients Unfit for Platinum-Based First-Line Therapy: Results of the MOON-OSS Observational Trial
Source: Cancers (Basel). 2022 Dec 9;14(24):6074. doi: 10.3390/cancers14246074 (PMC9776592; doi:10.3390/cancers14246074)
Supplement: Supplementary file 1 [file cancers-14-06074-s001.zip › cancers-2092290-supplementary.pdf]

Supplementary Table S1

Treatment	All grade	Grade 3-4	Dose delays	Dose reductions
**Gemcitabine**	63.7%	13.6%	41%	31.8%
**Oral standard vinorelbine**	66.7%	16.6%	16.6%	33.3%
**Metronomic vinorelbine**	65.9%	8%	13.8%	17.8%
**Others***	71.4%	14.3%	28.6%	28.6%

*Others group (n = 7) consisted of docetaxel and pemetrexed.
